# Intrinsic Peroxidase-like Activity of Ficin

**DOI:** 10.1038/srep43141

**Published:** 2017-02-22

**Authors:** Yufang Yang, Dongjun Shen, Yijuan Long, Zhixiong Xie, Huzhi Zheng

**Affiliations:** 1 The key Laboratory on Luminescent and Real-Time Analytical Chemistry, Ministry of Education, College of Chemistry and Chemical Engineering, Southwest University, Chongqing, 400715, P. R. China; 2 College of Life Sciences, Wuhan University, Wuhan, Hubei, 430072, P. R. China

## Abstract

Ficin is classified as a sulfhydryl protease isolated from the latex of fig trees. In most cases, a particular enzyme fits a few types of substrate and catalyzes one type of reaction. In this investigation, we found sufficient proofs for the intrinsic peroxidase-like activity of ficin and designed experiments to examine its effectiveness in a variety of scenarios. Ficin can transform peroxidase substrates to colored products in the existence of H_2_O_2_. Our results also indicate that the active sites of peroxidase-like activity of ficin are different from that of protease, which reveals that one enzyme may catalyze more than one kind of substrate to perform different types of reactions. On the basis of these findings, H_2_O_2_ releasing from MCF-7 cells was detected successfully. Our findings support a wider application of ficin in biochemistry and open up the possibility of utilizing ficin as enzymatic mimics in biotechnology and environmental monitoring.

Enzymes are indispensable biological catalysts for self-replication and for the metabolism of organisms. They are the most specific catalysts known^1^, both from the viewpoint of the substrate and the type of reaction performed on the substrate^2^. In most cases, a particular enzyme fits a few types of substrate and catalyzes one type of reaction. One exception is DNA polymerases, which are widely used in polymerase chain reaction (PCR), possess both polymerase activity and exonuclease activity. These different activities are often located in separately structured domains on the same polypeptide chain^3^,^4^. However, reports of enzymes catalyzing more than one type of reaction are very scarce.

Peroxidases (POXs, EC 1.11.1.x) are a large family of enzymes, found extensively in animals, plants, and microorganisms. Class III plant peroxidase (POX, EC 1.11.1.7), a plant-specific oxidoreductase and heme-containing glycoprotein^5^, plays a part in increasing the plant defenses against pathogens^6^. Peroxidase has a ferriprotoporphyrin IX prosthetic group located at the active site. The major hallmark of this kind of enzymes is the ability to catalyze H_2_O_2_-dependent oxidoreduction, and reduce the toxicity of peroxides and some aromatic compounds (electron donors)^7^. They can also catalyze the conversion of chromogenic substrates into colored products that are detectable by spectrophotometric methods. Therefore, they have been widely applied to biochemical analyses^8^,^9^, such as western-blots^10^, enzyme-linked immunoabsorbent assay^11^ and immunohistochemistry^12^.

Horseradish peroxidase (HRP, EC1.11.1.7) is one of the most important peroxidases used in biochemical analysis. However, the applications of HRP are still limited because of its rigorous storage requirements, poor thermal stability, high expense, sensitivity to the environment and its short storage life due to denaturation and digestion. As a consequence, there is a good deal of current research interest in artificial enzyme mimics. To date, more and more mimetic enzymes have put to use, such as metal-oxides nanoparticles^13^,^14^, heme complex^15^,^16^, graphene oxide^17^,^18^, ionic nanoparticles^19^, carbon nanodots^20^, quantum dots^21^, and metal-organic frameworks^22^. These mimetic enzymes overcome the drawbacks of HRP and promote the development of artificial enzyme mimics. To one’s disappointment, however, some of these non-biological catalysts often need laborious preparation procedures and modification steps to suppress aggregation, which would result in low reproducibility and low catalytic activity. Biological catalysts, in comparison to non-biological catalysts, possess particularly high catalytic efficiency, high reaction rates under very mild and favorable biological reaction conditions. For this reason, it is still highly desirable to find biological peroxidase-like materials.

Ficin (EC 3.4.22.3), isolated from the latex of fig trees, is classified as a sulfhydryl protease. It cleaves proteins at the carboxyl side of glycine, serine, threonine, methionine, lysine, arginine, tyrosine, alanine, asparagine and valine. Ficin contains eight cysteine, and is stabilized by three disulfide bridges^23^. It is generally recognized that cysteine and histidine play a key role in the residues for the protease activity of ficin^24^,^25^,^26^. The sequence of amino acids around active sites has high degree of homology with the corresponding one in the cysteine protease papain^27^. Here we show our discovery that ficin possesses intrinsic peroxidase-like activity. And our results indicate that the active sites of peroxidase-like activity of ficin are different from that of protease. Our findings reveal that one enzyme may catalyze more than one kind of substrates to perform different type of reactions. On the basis of these findings, H_2_O_2_ releasing from MCF-7 cells was detected successfully.

## Results and Discussion

### Discovery of intrinsic peroxidase-like activity of ficin

**Figure 1:**
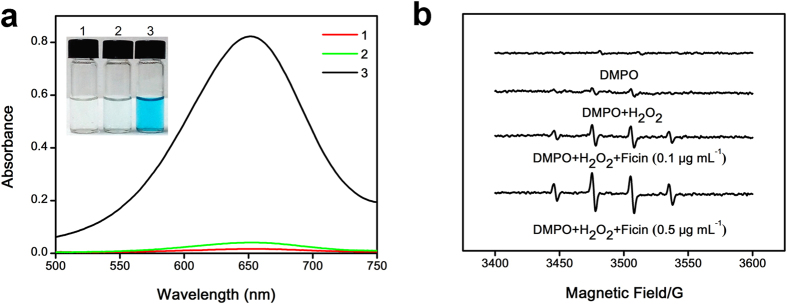
Ficin shows intrinsic peroxidase-like activity. (**a**) The absorption spectra of TMB in different reaction systems: TMB+Ficin (1), TMB + H_2_O_2_ (2), and TMB+H_2_O_2_ + Ficin (3) in 20 mM PBS buffer (pH 5.0) at 35 °C after 2 hour incubation. The concentrations were 0.10 μg mL^−1^ for ficin, 0.80 mM for H_2_O_2_ and TMB. The inset shows corresponding digital image. (**b**) EPR spectra of •OH radicals in the system of DMPO, DMPO-H_2_O_2_, DMPO-H_2_O_2_-Ficin (0.10 μg mL^−1^) and DMPO-H_2_O_2_-Ficin (0.50 μg mL^−1^).

Nature peroxidases, such as HRP, show strong catalytic activity and substrate specificity in the transformation of chromogenic substrates to colored products in the existence of H_2_O_2_^28^. Herein, we found sufficient proof for intrinsic peroxidase-like activity of ficin. As shown in Figure 1a, in different reaction systems, 3,3′,5,5′-tetramethylbenzidine (TMB), the typical substrate for peroxidases, was oxidized in the existence of H_2_O_2_ only when ficin was added, resulting in a blue reaction product and a maximum absorbance at 652 nm^29^. This result showed that the peroxidase-like activity toward TMB came from the robust intrinsic catalytic property of ficin. To further verify the peroxidase-like activity of ficin, we performed experiments using other peroxidase substrates instead of TMB, including o-phenylenediamine (OPD) and 2,2′-azino-bis (3-ethylbenzthiazo-line-6-sulfonic acid) diammonium salt (ABTS). We found that ficin catalyzed the oxidation of TMB, OPD and ABTS by H_2_O_2_ in pH 5.0 PBS buffer, followed the expected typical color changes (Figure S1). By comparison, ficin or H_2_O_2_ alone could not produce significant color change. These results confirm that ficin possesses the capability to catalyze oxidation of organic substrates and exhibits an intrinsic peroxidase-like activity.

The intrinsic peroxidase-like activity of ficin can be further certified by the EPR experiment. 5,5-Dimethyl-1-pyrroline-N-oxide (DMPO), a widely used hydroxyl radical trapping reagent, is applied to confirm the generation of DMPO/•OH spin adduct with high sensitivity and selectivity. As Fig. 1b shows, the typical DMPO/•OH spin adduct signal intensity in the systems of DMPO and DMPO-H_2_O_2_ are not significant when compared to DMPO-H_2_O_2_-ficin, demonstrating that ficin converts H_2_O_2_ to •OH radical^29^. In addition, the typical DMPO/•OH spin adduct signal intensity was increased by the increase in the concentration of ficin, demonstrating that the peroxidase-like activity of ficin may originate from the catalytic ability to convert H_2_O_2_ to •OH radical. The results of EPR provide empirical evidence that ficin possesses intrinsic peroxidase-like activity.

### pH, temperature, substrate concentrations, and incubation time dependence

**Figure 2:**
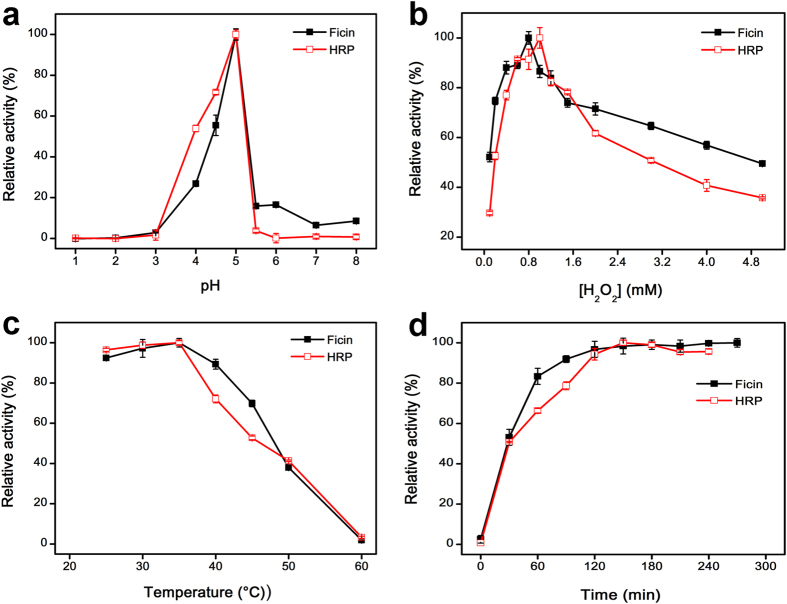
The peroxidase-like activity of ficin is pH (**a**), H_2_O_2_ concentration (**b**), temperature (**c**), and incubation time (**d**) dependent. Experiments were carried out using 0.10 μg mL^−1^ ficin or 0.10 ng mL^−1^ HRP with 0.80 mM TMB as substrate, respectively. The H_2_O_2_ concentration was 0.80 mM for ficin and 1.0 mM for HRP. The pH was 5.0, and the temperature was 35 °C unless otherwise stated. For each curve, the maximum point was defined as 100% and error bars represent the standard deviations of three independent experiments.

Similar to HRP, the peroxidase-like activity of ficin is also dependent on experimental conditions. We explored the effect of pH, temperature, H_2_O_2_ concentration, TMB concentration and reaction time on the relative activity (Fig. 2 and Figure S2). At a weakly acidic pH, ficin possesses higher catalytic activity, and the maximal relative activity of ficin was found at pH 5.0 (Fig. 2a). The effect of H_2_O_2_ concentration on relative activity was tested in the range of 0.10 to 5.0 mM, and the relative activity increased with an increasing H_2_O_2_ concentration in the range from 0.10 to 0.80 mM. With a concentration of H_2_O_2_ higher than 0.80 mM, the relative activity decreased (Fig. 2b). Higher H_2_O_2_ concentrations will inhibit the peroxidase-like activity of ficin, and this behavior is similar to HRP^30^. As shown in Fig. 2c and d, the optimal temperature and reaction time for the maximum relative activity of ficin were 35 °C and 2 hours. The effect of the concentration of TMB was also investigated. As shown in Figure S2, the optimal TMB concentration for maximum relative activity of ficin was 0.80 mM. Thus, the optimal pH, temperature, H_2_O_2_ concentration, TMB concentration and reaction time were as follows: 5.0, 35 °C, 0.80 mM, 0.80 mM, 2 hours. These optimum parameters were very similar to those observed with HRP, but different from those of protease^31^.

### Michaelis constant determination and catalytic mechanism

**Figure 3:**
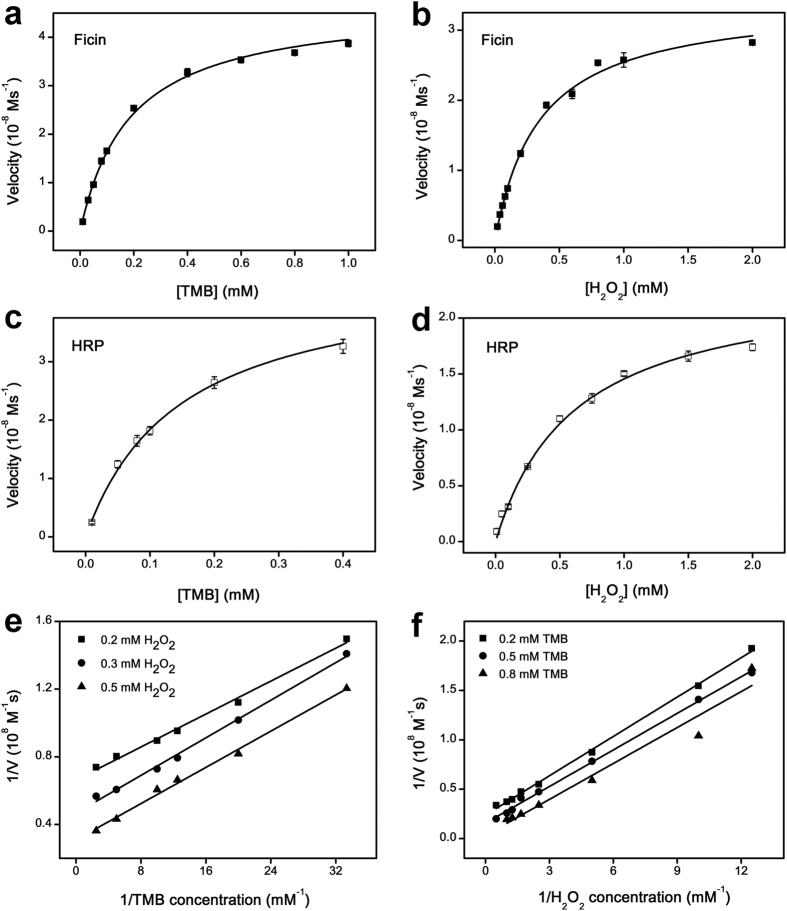
Steady-state Kinetic Assay and Catalytic Mechanism of Ficin. (**a–d**) The velocity (*v*) of the reaction was measured using 1.0 μg mL^−1^ ficin (**a,b**) or 1.0 ng mL^−1^ HRP (**c,d**) under optimal conditions. Error bars represent the standard deviations of three independent experiments. (**a,c**) The concentration of H_2_O_2_ was 0.80 mM (ficin) or 1.0 mM (HRP) and varied concentration of TMB. (**b,d**) The concentration of TMB was 0.40 mM (ficin) or 0.60 mM (HRP) and varied concentration of H_2_O_2_. (**e**,**f**) Double-reciprocal plots of activity of ficin at a fixed concentration of one substrate versus varying concentration of the second substrate.

Aiming at measuring the catalytic mechanism of ficin, the steady-state kinetic parameters for the oxidation of TMB in the presence of H_2_O_2_ were measured, and results were placed within a Michaelis-Menten kinetic model^32^. Within the suitable range of substrate (TMB and H_2_O_2_), typical Michaelis-Menten curves were obtained for both ficin (Fig. 3a,b) and HRP (Fig. 3c,d). The data were fitted well to the Michaelis-Menten model to acquire the enzyme kinetic parameters (Michaelis constant, maximum reaction rate, catalytic constant and catalytic efficiency) summarized in Table 1. We obtained the *K*_m_ (TMB) of 0.19 mM, and the *K*_m_ (H_2_O_2_) of 0.35 mM. Thus, the *K*_m_ (TMB) for ficin was similar to that of HRP (0.15 mM), while the *K*_m_ (H_2_O_2_) for ficin was 1.74 times lower than that of HRP (0.61 mM), suggesting that ficin has a higher affinity to H_2_O_2_. In addition, the catalytic efficiency (*K*_cat_/*K*_m_) of ficin was calculated to be 5.89 mM^−1^ s^−1^ for TMB, and 2.31 mM^−1^ s^−1^ for H_2_O_2_. The catalytic efficiency of ficin were approximately 3 orders of magnitude lower than HRP. It may be due to the fact that ficin is a simple enzyme while HRP is a conjugated enzyme containing the ferroprotoporphyrin group.

To further analyze the catalytic mechanism of ficin, double reciprocal plots of initial velocity catalytic mechanism were measured. Figure 3e and f show double reciprocal plots of initial velocity against one substrate concentration, obtained in a range of concentrations of the second substrate. Similar to HRP, the lines are mutually parallel, which is the characteristic of a Ping-Pong mechanism^33^. This finding clearly demonstrates that the catalytic mechanism of ficin is that ficin binds and reacts with the first substrate, then releases the first product before reacting with the second substrate.

### Comparison of robustness of peroxidase-like activity of ficin and HRP

**Figure 4:**
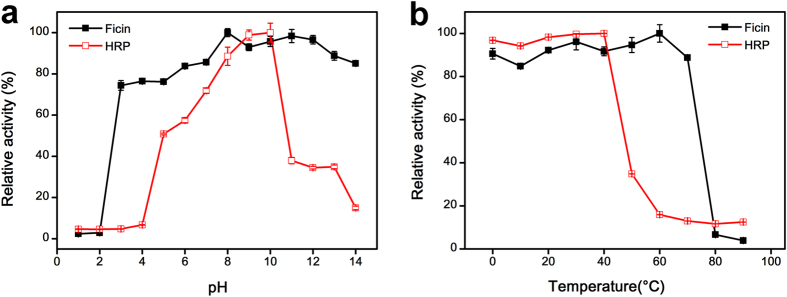
Effects of pH (**a**) and temperature (**b**) on the robustness of ficin and HRP. 1.0 mg mL^−1^ ficin and 1.0 μg mL^−1^ HRP were first incubated at pH 1–14 for 2 h or 0–90 °C for 2 h. And then they were diluted to 0.10 μg mL^−1^ (ficin) or 0.10 ng mL^−1^ (HRP) to test the peroxidase activities under optimal conditions (20 mM PBS buffer, pH 5.0, 35 °C, 0.80 mM H_2_O_2_ and TMB). For each curve, the maximum point was defined as 100% and error bars represent the standard deviations of three independent experiments.

To investigate the pH and thermal robustness of peroxidase-like activity of ficin, ficin was first incubated in solution at a pH range (1–14) or a range of values of temperature (0–90 °C) for 2 hours. The peroxidase activity was tested under optimal conditions (pH 5.0 and 35 °C). For comparison, the catalytic activity of HRP was tested under same conditions, and the results were shown in Fig. 4a and b. Ficin showed robust peroxidase activity over a range of pH from 3.0 to 14.0, while the catalytic activity of HRP significantly decreased when the pH was lower than 4.0 or higher than 11.0, which is essentially in agreement with previous reports^15^,^17^. Meanwhile, ficin was found to possess outstanding thermal stability over a wide range of temperature from 0 to 70 °C, while the activity of HRP decreased by 70% over 50 °C. What is noteworthy is that this outstanding robustness of peroxidase-like activity of ficin against harsh pH and temperature suggest that it can be used more extensively in analytic applications than HRP.

### Peroxidase-like activity origins from ficin not impurities

It’s important to confirm that the observed peroxidase-like activity originated from ficin itself rather than the impurities coexisting in it. To rule out the possibility that the observed activity was caused by the peroxidase existing in ficin, highly purified premium grade ficin was used in our experiments. To further confirm it, 2× crystallized ficin were also used. After the removal of cysteine, which was added by the supplier to activate the protease activity of ficin, 2× crystallized ficin can also catalyze the oxidation of TMB by H_2_O_2_ (Figure S3). These results show that the peroxidase-like activity is not due to coexisting peroxidase. The robustness of peroxidase-like activity of ficin against harsh pH and temperature was also a collateral to support this conclusion.

Some metal ions can catalyze the chromogenic reaction between TMB and H_2_O_2_. To test the metal content of ficin, ICP-MS (Agilent 7700ce) was used. As illustrated in Table S1, the metal concentrations were less than 10 nM: Al (9.63 nM), Mg (9.17 nM), Zn (7.69 pM), Ca (2.15 nM), Fe (0.04 nM), K (2.08 nM). Additionally, we tested the catalytic effect of segmental metal ions coexisting in ficin under standard conditions (0.20 M PBS buffer, pH 5.0, 35 °C). It was found that the catalytic activity of metal ions (100 nM, 10 nM, 1 nM) was insignificant compared with ficin (Figure S4a), which supports our suggestion that the peroxidase-like activity originates from ficin rather than from the metal ions coexisting in ficin. Additionally, ethylenediaminete-traacetic acid (EDTA) was added into the reaction solution. Even though EDTA was added at concentrations up to 1.0 mM, the peroxidase-like activity of ficin did not change (Figure S4b), which further rules out the effect of metal ions. These results verified the peroxidase-like activity is due to ficin not impurities.

### Effects of mercuric chloride and iodoacetic acid on the peroxidase-like activity of ficin

A reduced -SH group is necessary for the protease activity of ficin^34^, and this activity can be inhibited by mercuric chloride and iodoacetic acid due to the binding to cysteine^35^, one of the active sites of ficin when it acts as a protease^27^. Herein, we tested effects of mercuric chloride and iodoacetic acid on the peroxidase-like activity of ficin. However, neither 100 μM of mercuric chloride nor 100 μM of iodoacetic acid inhibited the peroxidase-like activity of ficin (Figure S5). This result indicates that the active sites of peroxidase-like activity are different from that of protease.

As a protease, ficin contains about 200 amino acids, and its catalytic dyad is made up of cysteine and histidine. Why did ficin develop a polypeptide chain with more amino acids than necessary during the long-term natural evolution? Except in the disulfide bridges and active sites, what is the function of other amino acids? Peroxidases are known to play a part in increasing a plant’s defenses against pathogens and reducing the toxicity of some organic substrates^6^. Hence, it is logical to discover that a protease possesses peroxidase-like activity.

### Ficin applied to detect the H_2_O_2_ releasing from living cells

**Figure 5:**
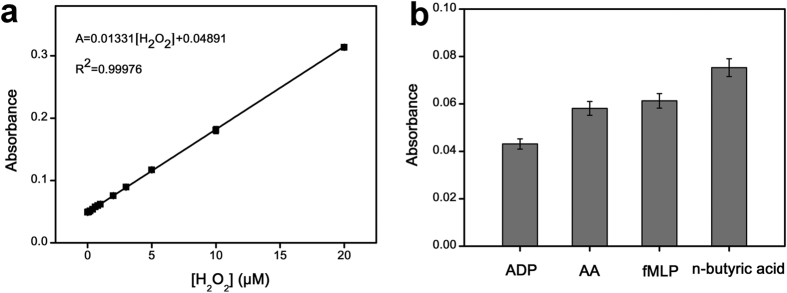
Ficin applied to detect the H_2_O_2_ release from MCF-7 cells. (**a)** Linearity of absorbance against a H_2_O_2_ concentration range of 0–20 μM. (**b**) Comparison of fluxes of H_2_O_2_ release from cells induced by ADP (200 ng mL^−1^, 0.47 μM), AA (200 ng mL^−1^, 1.14 μM), fMLP (200 ng mL^−1^, 0.46 μM) and n-butyric acid (10 mM). All the conditions: 0.10 μg mL^−1^ ficin, 0.80 mM TMB, 20 mM PBS buffer (pH 5.0), and 35 °C. Error bars represent the standard deviations of three independent experiments.

The color variation of TMB oxidation catalyzed by ficin was H_2_O_2_ concentration-dependent. This indicates that the absorbance change can be used for the detection of H_2_O_2_ (Fig. 5a). Based on the peroxidase-like activity of ficin, H_2_O_2_ releasing from MCF-7 cells was detected successfully (Fig. 5b). When the MCF-7 cells were stimulated by 10 mM n-butyric acid, 1.96 μΜ H_2_O_2_ releasing occurred, calculated to be 9.80 × 10^−15^ mol/cell. This value matches with that reported previously^36^,^37^. To evaluate effects of other stimuli, the amount of H_2_O_2_ releasing from MCF-7 cells induced by adenosine-5-diphosphate (ADP, 200 ng mL^−1^), ascorbic acid (AA, 200 ng mL^−1^), n-formylmethionyl-leucylphenyl-alanine (fMLP, 200 ng mL^−1^) were also measured. As shown in Fig. 5B, the highest amount of H_2_O_2_ releasing occurred by using 10 mM n-butyric acid as stimuli.

## Conclusions

In summary, we found sufficient proofs for the intrinsic peroxidase activity of ficin, which catalyzed the reaction of different peroxidase substrates in the presence of H_2_O_2_. In addition, the active sites of peroxidase-like activity of ficin are different from those of protease, which reveals that one enzyme may catalyze more than one kind of substrate to perform different types of reactions. The peroxidase-like activity of ficin was dependent on pH, temperature, H_2_O_2_ concentration, incubation time, and showed typical Michaelis-Menten kinetics with a Ping-Pong mechanism. Based on the peroxidase-like activity of ficin, H_2_O_2_ releasing from MCF-7 cells was detected successfully. We argue that the peroxidase-like activity of ficin may play a part in defending pathogens and reducing toxicity of some organic compounds. Our findings support a wider application of ficin in biochemistry and open up the possibility of utilizing ficin as enzymatic mimics in biotechnology and environmental monitoring.

## Methods

### Reagents and materials

3,3′,5,5′-tetramethylbenzidine (TMB), o-phenylenediamine (OPD) and 2,2′-azino-bis (3-ethylbenzo-thiazoline-6-sulfonic acid) diammonium salt (ABTS) were obtained from Sangon Biotech Co. Ltd. Premium grade ficin (F4165, powder, molecular weight 23.8 kDa), 2× crystallized ficin (F4125, saline suspension, containing 30 mM cysteine) and horseradish peroxidase (HRP, 300 U mg^−1^) were purchased from Sigma-Aldrich. H_2_O_2_ was obtained from Chongqing Pharmaceutical Co., Ltd. N-butyric acid, adenosine- 5-diphosphate (ADP), ascorbic acid (AA), and n-formylmethionyl-leucyl-phenylalanine (fMLP) were obtained from Aladdin. Ultrapure water (18.2 MΩ) was prepared with a Milli-Q system and used in all experiments. A NaH_2_PO_4_-Na_2_HPO_4_ buffer solution (PBS, 20 mM, pH from 1.0–12.0) was used in this study, and the pH of buffer solutions was adjusted with H_3_PO_4_ or NaOH (20 mM) solution. Additionally, for pH 13 or 14, KOH solutions were used to replace corresponding buffer solutions.

### Electron paramagnetic resonance (EPR)

Samples were prepared at room temperature by adding 4.0 mM H_2_O_2_, and 25.0 mM DMPO with different concentrations of ficin, and then adding a 20 mM PBS buffer (pH = 5.0) into a plastic tube. Subsequently the prepared sample solution was transferred to a quartz capillary tube and placed in the EPR cavity. Spectra were recorded afterwards. DMPO was used to trap the •OH radicals to form the DMPO/•OH spin adduct. The EPR spectra were obtained on a JESFA200 (JEOL, Japan) with a microwave bridge (modulation width, 0.2 mT; modulation amplitude, 2 Gauss; frequency power, 1 mW; modulation frequency, 100 kHz).

### Kinetic analysis

Steady-kinetic measurements were carried out by monitoring the change in absorbance at 652 nm on a microplate reader (Infinite 200 PRO, TECAN, Austria). Experiments were carried out using 1.0 μg mL^−1^ ficin or 1.0 ng mL^−1^ HRP in 20 mM PBS buffer (pH 5.0) at 35 °C with 0.80 mM H_2_O_2_ for ficin, 1.0 mM for HRP or 0.40 mM TMB for ficin, 0.60 mM for HRP as substrate, unless otherwise stated. Double-reciprocal plots of activity of ficin were obtained at a fixed concentration of one substrate versus varying concentrations of the second substrate. The Michaelis–Menten constant was calculated using Lineweaver–Burk plots of the double reciprocal of the Michaelis−Menten equation, 1/*ν* = *K*_m_/*V*_max_⋅(1/[S]+1/*K*_m_), where *v* is the initial velocity, *V*_max_ is the maximal reaction velocity, [S] is the concentration of substrate, *K*_m_ is the Michaelis constant.

All other spectroscopy measurements were carried out by recording the absorbance change at 652 nm with a UV-Vis spectrophotometer (UV-2450, Shimadzu, Japan).

### Removal of cysteine existing in 2×crystallized ficin

To active the protease activity of ficin, 30 mM of cysteine was added in 2× crystallized ficin by the supplier. However, cysteine can inhibit the peroxidase-like activity of ficin drastically. Therefore, we removed cysteine by centrifugal ultrafiltration or dialysis before investigating the peroxidase-like activity of 2× crystallized ficin. They were both suspended in a 10-fold dilution saline suspension. Then the exact concentration of ficin was estimated based on percentile absorptivity E^1%^ = 21.0 (280 nm). Centrifugal ultrafiltration (ultra-filtration tubes with cutoff molecular weight of 5 kDa) was carried out at 4 °C, 3,000 rpm for 10 minutes each time and repeated 10 times just before use. The stock solution of ficin was dialyzed (dialysis bags with cutoff molecular weight of 5 kDa) in ultra-pure water for 48 hours at 4 °C and the ultra-pure water was changed every two hours.

### Culture of MCF-7 cell

MCF-7 cell (human breast cancer cell) lines purchased from the Type Culture Collection of the Chinese Academy of Sciences (Shanghai) were used as target cells. Cells were incubated in DMEM containing 10% fetal bovine serum (FBS, Gicbo), 100 U mL^−1^ of penicillin and 100 μg mL^−1^ streptomycin. They were maintained at 37 °C in a humidified and 5% CO_2_ incubator. The cell density was determined using a Scepter handheld automated cell counter (Millipore), and this was performed prior to experiments.

### Measurement procedure of H_2_O_2_

For measuring the H_2_O_2_ standard solution, the H_2_O_2_ concentration-dependent absorbance was studied. In a typical process, 0.10 μg mL^−1^ ficin and 0.80 mM TMB were added in 96-well plate, after which several different concentrations of H_2_O_2_ was added, and then the above mixture was incubated in 20 mM PBS buffer (pH 5.0) at 35 °C for 2 hours to allow development of the blue color. Next, the absorbance at 652 nm of each well was determined immediately by a microplate reader (Infinite 200 PRO, TECAN, Austria).

To measure the H_2_O_2_ releasing from living cells, MCF-7 cancer cell was cultured in 96-well plate with cell density of 2.0 × 10^5^ cells mL^−1^ for 24 hours. After the cultural medium was removed, 10 mM n-butyric acid was added, and this mixture was incubated for 1 min by shaking. Then 0.10 μg mL^−1^ ficin, 0.80 mM TMB, 20 mM PBS buffer (pH 5.0) were further added (a final volume of 200 μL) and incubated at 35 °C for 2 hours to finish the reaction. The absorbance was determined by a microplate reader. H_2_O_2_ releasing from MCF-7 cells induced by ADP (200 ng mL^−1^, 0.47 μM), AA (200 ng mL^−1^, 1.14 μM), fMLP (200 ng mL^−1^, 0.46 μM) were also measured, and compared with those induced by n-butyric acid (the number of cells used in the measurements is ~4 × 10^5^ cells).

## Additional Information

**How to cite this article**: Yang, Y. *et al*. Intrinsic Peroxidase-like Activity of Ficin. *Sci. Rep.* **7**, 43141; doi: 10.1038/srep43141 (2017).

**Publisher's note:** Springer Nature remains neutral with regard to jurisdictional claims in published maps and institutional affiliations.

## Supplementary Material

Supplementary Information

## Figures and Tables

**Table 1:** Comparison of the kinetic parameters between Ficin and HRP.

Enzyme	[*E*] (M)	Substance	*K*_m_ (mM)^a^	*V*_max_ (10^−8^ M s^−1^)^a^	*K*_cat_ (s^−1^)	*K*_cat_/*K*_m_ (mM^−1^ s^−1^)
Ficin	4.20 × 10^−8^	TMB	0.19 ± 0.012	4.69 ± 0.15	1.12	5.89
Ficin	4.20 × 10^−8^	H_2_O_2_	0.35 ± 0.017	3.42 ± 0.085	0.81	2.31
HRP	2.27 × 10^−11^	TMB	0.15 ± 0.018	4.53 ± 0.30	2.00 × 10^3^	13.3 × 10^3^
HRP	2.27 × 10^−11^	H_2_O_2_	0.61 ± 0.042	2.35 ± 0.087	1.04 × 10^3^	1.70 × 10^3^

^a^Mean value ± standard deviation (three independent experiments).

[*E*] is the enzyme concentration, *K*_m_ is the Michaelis constant, *V*_max_ is the maximum reaction rate, *K*_cat_ is the catalytic constant, where *K*_cat_ = *V*_max_/[*E*], and *K*_cat_/*K*_m_ is the catalytic efficiency.
