# Supplementary material for: Intrinsic Peroxidase-like Activity of Ficin
Source: Sci Rep. 2017 Feb 22;7:43141. doi: 10.1038/srep43141 (PMC5320487; doi:10.1038/srep43141)
Supplement: Supplementary Information [file srep43141-s1.pdf]

Supporting Information:

Intrinsic Peroxidase-like Activity of Ficin

Yufang Yang[a], Dongjun Shen[a], Yijuan Long[a], Zhixiong Xie[b] & Huzhi Zheng[a1*]

[a]The key Laboratory on Luminescent and Real-Time Analytical Chemistry, Ministry of Education, College of Chemistry and Chemical Engineering, Southwest University, Chongqing 400715, P. R. China. Fax: +86-23-68254000

E-mail: zhenghz@swu.edu.cn (H. Zheng)

[b]College of Life Sciences, Wuhan University, Wuhan, Hubei, 430072, P. R. China.

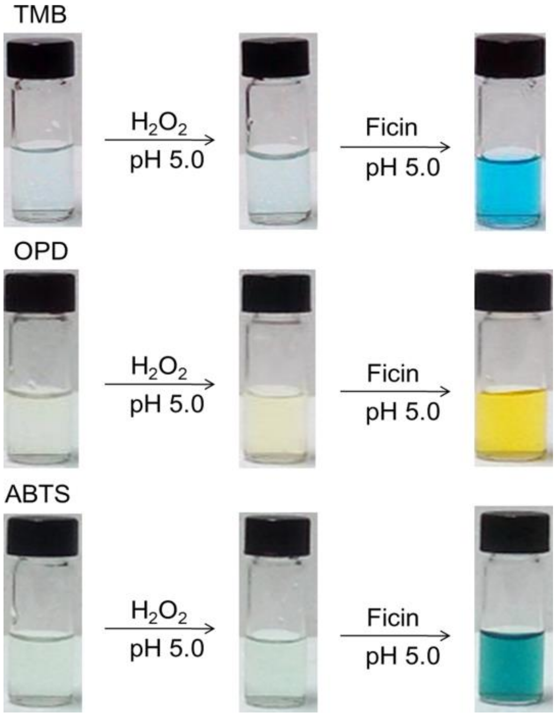

Fig. S1 Ficin shows peroxidase-like activity. Experiments were carried out using 0.10 μg mL^{-1} ficin with 0.80 mM TMB, 1.0 mM H_2O_2, and 0.50 μg mL^{-1} ficin with 5.0 mM OPD or ABTS, 0.50 mM H_2O_2, 20 mM PBS buffer (pH 5.0) at 35 ℃ after 2 h incubation.

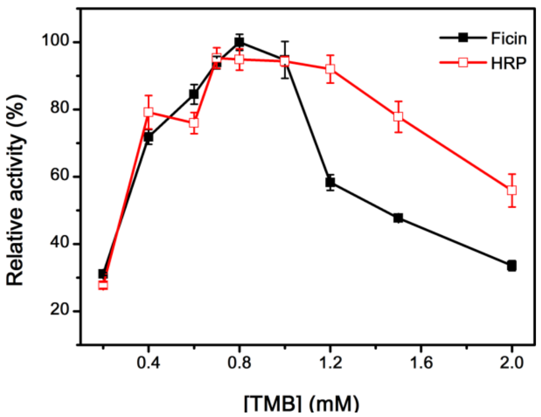

Fig. S2 The peroxidase-like activity of ficin is TMB concentration dependent. Experiments were carried out using 0.10 μg mL^{-1} ficin or 0.10 ng mL^{-1} HRP. The H_2O_2 concentration was 0.80 mM for ficin and 1.0 mM for HRP. The pH was 5.0, and the temperature was 35 ℃ unless otherwise stated. For each curve, the maximum point was defined as 100% and error bars represent the standard deviations of three independent experiments.

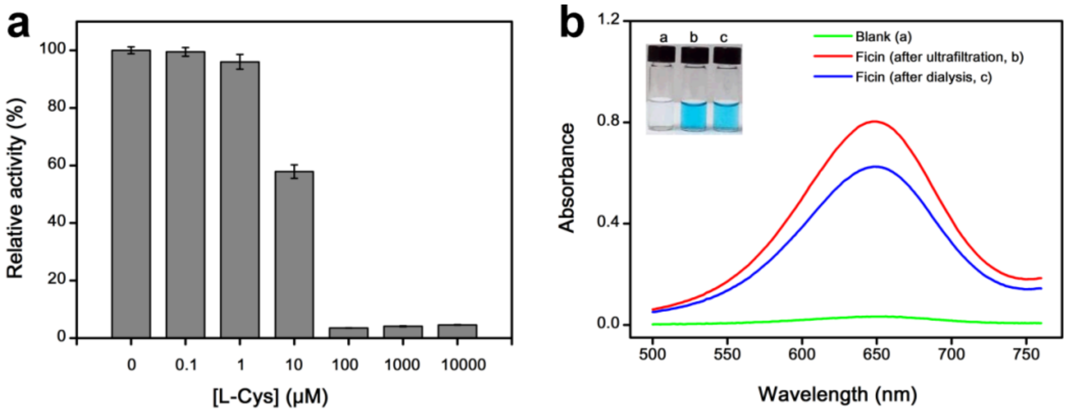

Fig. S3 2× Crystallized ficin possesses intrinsic peroxidase-like activity. a. The effect of L-cysteine on the peroxidase-like activity of ficin. The concentration of ficin was 0.10 μg mL^{-1}. b. 2× Crystallized ficin possesses peroxidase-like activity. The inset shows corresponding digital image. The concentration of 2× crystallized ficin was 0.10 mg mL^{-1}, and assays were carried out under standard conditions (20 mM PBS buffer, pH 5.0, 35 ℃, 0.80 mM TMB and H_2O_2). Error bars represent the standard deviations of three independent experiments.

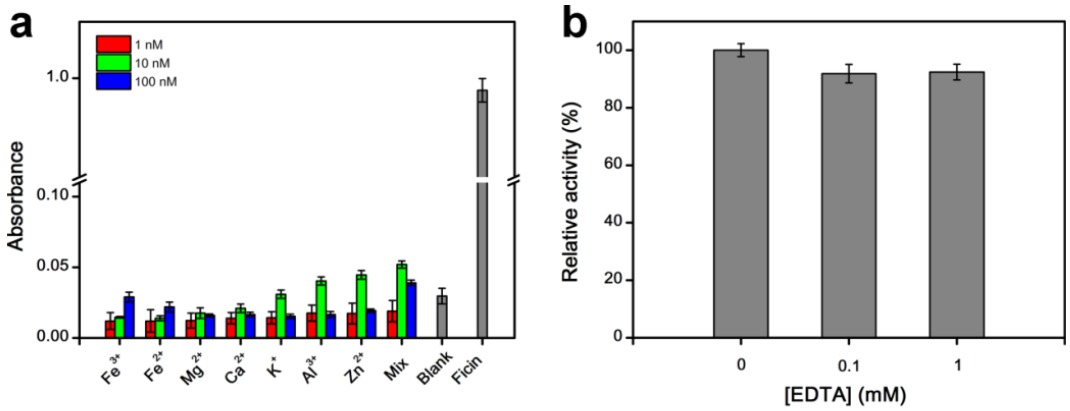

Fig. S4 Effect of metal ions on the peroxidase-like activity of ficin. a. The catalytic activity of segmental metal ions coexisting in ficin. b. The effect of EDTA on the peroxidase-like activity of ficin. The metal ions concentrations were 1 nM, 10 nM and 100 nM. The concentration of ficin was 0.10 μg mL^{-1}. Assays were carried out under standard conditions (20 mM PBS buffer, pH 5.0, 35 ℃, 0.80 mM H_2O_2 and TMB). Error bars represent the standard deviations of three independent experiments.

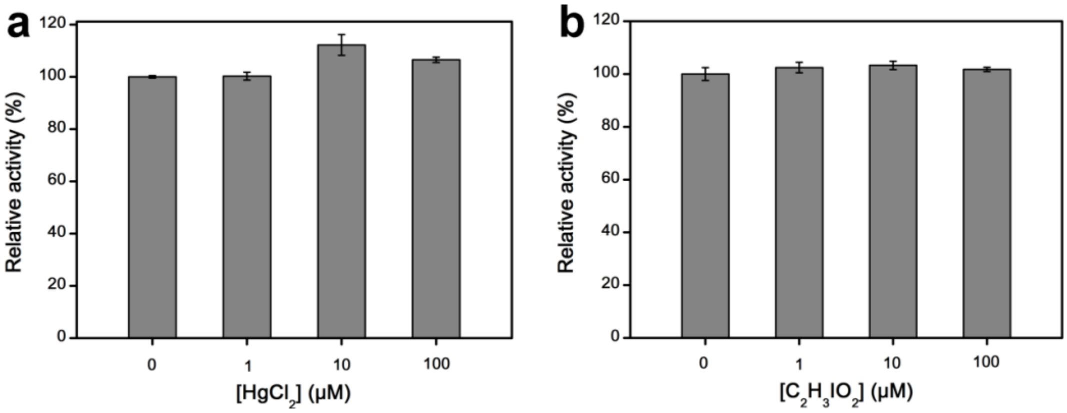

Fig. S5 Effects of mercuric chloride (a) and iodoacetic acid (b) on the peroxidase-like activity of ficin. The incubation time of ficin with mercuric chloride or iodoacetic acid is 30 min and the concentration of ficin is 0.10 μg mL^{-1}. The experiment was carried out under the optimal conditions (20 mM PBS buffer, pH 5.0, 35 ℃, 0.80 mM H_2O_2 and TMB). The peroxidase-like activity of ficin in the absence of mercuric chloride or iodoacetic acid was defined as 100%. Error bars represent the standard deviations of three independent experiments.

Table S1. Metal content of ficin (determined by ICP-MS)

Metal	Content (wt %)	Metal	Content (wt %)	Metal	Content (wt %)
Al	0.26	Na	0.42	Mn	< 0.0001
Mg	0.22	Be	< 0.0001	Sn	< 0.0001
Zn	0.0005	Pb	< 0.0001	Bi	< 0.0001
Ca	0.086	As	< 0.0001	Co	< 0.0001
Fe	0.0023	Ti	< 0.0001	Ni	< 0.0001
K	0.081	V	< 0.0001	Cu	< 0.0001
